# Fetal dose in pregnant CT patients: a comparison of four software packages

**DOI:** 10.1007/s00330-025-11594-1

**Published:** 2025-04-25

**Authors:** Joël Greffier, Asma Arjoun, Chris Serrand, Jean-Paul Beregi, Djamel Dabli

**Affiliations:** 1 IMAGINE UR UM 103, Montpellier University, Department of Medical Imaging, Nîmes University Hospital, Nîmes, France; 2 Department of Biostatistics, Epidemiology, Public Health and Innovation in Methodology, Nîmes University Hospital, University of Montpellier, Nîmes, France

**Keywords:** Pregnant woman, Dosimetry, Radiation, Software^2^, Multidetector computed tomography

## Abstract

**Objectives:**

To compare the fetal dose (FD) as calculated by four different software packages for pregnant women who have undergone CT acquisitions directly exposing the whole fetus to X-rays.

**Materials and methods:**

Pregnant women who underwent CT abdomen–pelvis and/or thorax–abdomen–pelvis acquisitions from February 2018 to May 2024 and for whom the uterine dose and/or FD was calculated by a medical physicist were retrospectively included. FDs were computed per CT acquisition with VirtualDose-CT™ (VDCT), Duke Organ Dose (DOD), fetaldose.org, and COnceptus Dose Estimation (CODE) software, using phantoms taking the stage of pregnancy into account. FDs calculated by each software package were then compared.

**Results:**

A total of 51 pregnant women with a mean age of 30.2 ± 5.7 years at 17.5 ± 10.0 weeks of pregnancy were included. The mean number of CT acquisitions per pregnant patient was 1.4 ± 0.7 with a mean CTDI_vol_ of 6.77 ± 3.04 [2.34–15.64] mGy, and FDs were computed for a total of 69 acquisitions. For all CT acquisitions, the median FD was 8.6 (6.8; 10.3) mGy for VDCT, 7.7 (6.1; 9.7) mGy for DOD, 6.3 (4.9; 7.6) mGy for fetaldose.org, and 7.1 (4.6; 8.8) mGy for CODE. Differences between each software package were significant (*p* < 0.01), except between VDCT and DOD (*p* = 0.025) and between CODE and fetaldose.org (*p* = 0.15). The concordance of calculated FD values between the software packages was poor (ICC < 0.50), except between VDCT and CODE and between fetaldose.org and CODE.

**Conclusion:**

The choice of software used affects the calculation of the FD.

**Key Points:**

***Question**** Differences between calculation software in terms of morphologies and types of phantoms used have an impact on FD calculations*?

***Findings*** *Software choice has an impact on calculated FD, but is not expected to alter patient management except for extreme cases with multiple CT exams*.

***Clinical relevance*** *The FD limit of 100 mGy, defined by the International Commission on Radiological Protection, cannot be reached with a single CT examination, and may only be of concern in cases where the patient undergoes multiple exams with the whole fetus exposed*.

**Graphical Abstract:**

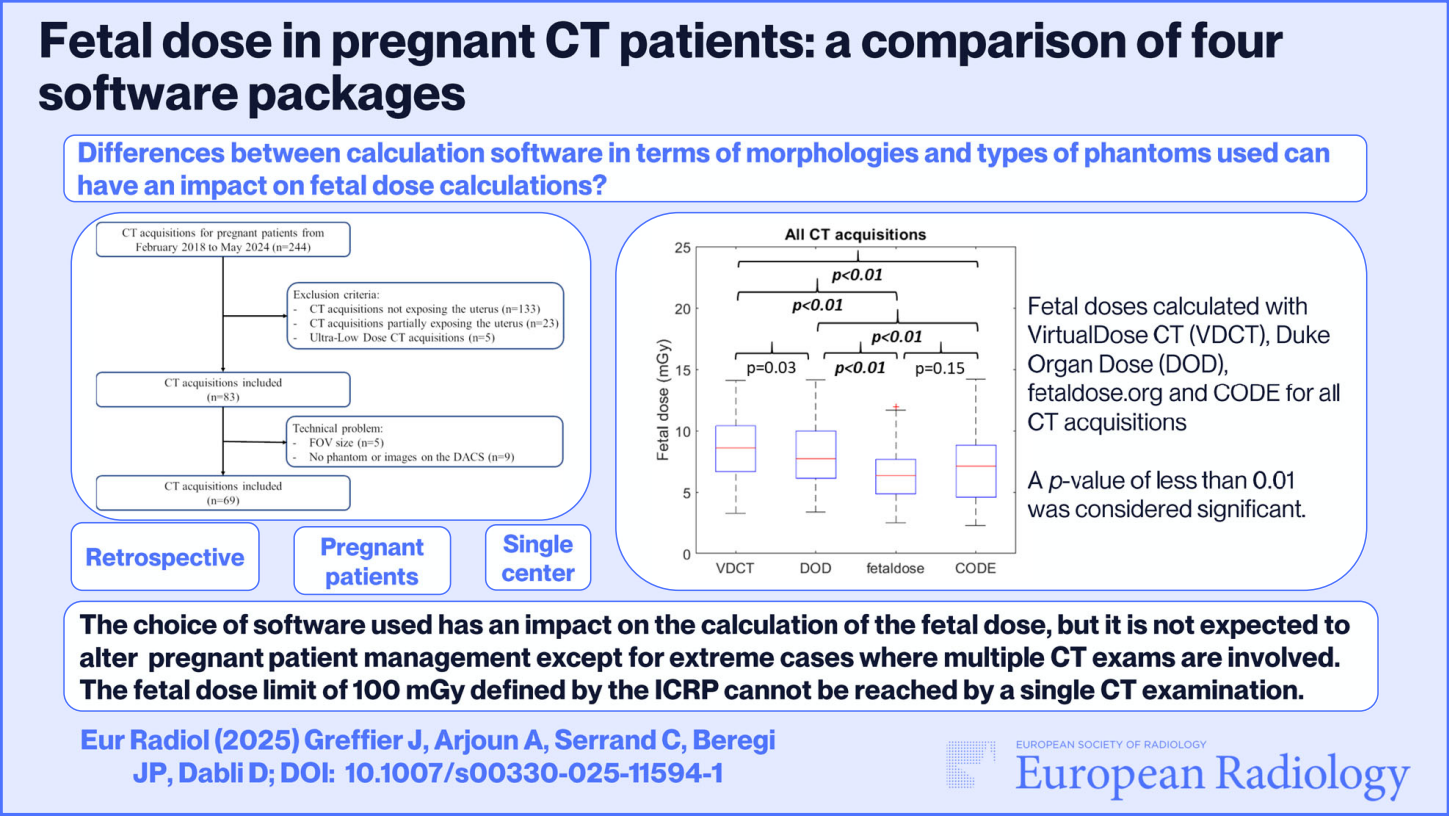

## Introduction

Throughout pregnancy, women can benefit from imaging procedures, particularly computed tomography (CT) examinations for abdominal pain, pulmonary embolism, trauma, etc. [1–3]. However, pregnant patients are considered to be at risk of exposure to X-rays [4–6]. The risk of stochastic (e.g., cancer) and teratogenic effects on the fetus (such as congenital malformations, impairment of mental development, and growth restrictions), depends on the gestational age and radiation dose absorbed [4, 5, 7–9]. Based on these effects, the International Commission on Radiological Protection (ICRP) has defined that fetal doses below 100 mGy should not be considered a reason to justify termination of pregnancy on the basis of radiological risk alone [5, 6, 10]. At fetal doses above this level, informed decisions should be made based upon individual circumstances.

For CT examinations, the dose delivered to the uterus or fetus will depend on the CT protocol used (standard, low-dose, or ultra-low-dose) and the position of the area exposed to radiation in relation to the position of the fetus [6, 11]. The more dose-optimized the CT protocol, the lower the dose to the uterus or fetus. The greater the size of the uterus or fetus exposed to radiation, the higher the dose to the uterus or fetus. Dabli et al showed that doses to the uterus were highest when the pelvis was directly exposed to X-rays, such as abdomen–pelvic or thorax–abdomen–pelvic CT acquisitions [11]. Consequently, it is essential to estimate the uterine and/or fetal dose (FD) when the pelvis is directly exposed, to assess the risk. To consider the advancement of the fetal stage, it is therefore preferable to calculate the FD and to use the appropriate calculation software.

Dedicated software packages are used to obtain the FD for each CT acquisition and assess the risk to the fetus. Each software package uses different phantoms and different FD calculation methodologies, and has its own advantages and limitations [5, 10, 12–24]. The main differences between these software packages lie in the morphologies and types of phantoms available to best adapt to the stage of pregnancy and the morphology of the patients exposed [10, 13–21]. Few studies have compared the differences calculated by the different calculation software packages available regarding the dose of radiation delivered to the fetus [25].

The aim of the present study was to compare the doses delivered to the fetus as calculated by four different dedicated software packages for pregnant women undergoing CT scans directly exposing the fetus.

## Materials and methods

### Patients

Data were collected retrospectively for pregnant patients who had undergone a CT examination at our institution and for whom the uterine and/or FD was calculated by a medical physicist with CT Expo from February 2018 to March 2023 and with VirtualDose-CT from March 2023 to May 2024. This retrospective single-center study was approved by our institutional review board (IRB#: 22.01.14), and the requirement for written informed consent was waived.

All pregnant patients for whom the uterus—and, therefore, the fetus—were directly and completely exposed to X-rays, such as abdomen–pelvis and thorax–abdomen–pelvis CT acquisitions, were included. Criteria for non-inclusion were CT acquisitions that did not expose, or only partially exposed, the fetus, such as chest, abdomen, chest–abdomen, lumbar spine, pelvis, etc. Ultra-low-dose CT acquisitions were also excluded, since the FD is minimal and not a dosimetric issue [11].

### CT examinations

Abdomen–pelvis and thorax–abdomen–pelvis CT examinations were performed using two different CT systems, a Somatom Definition Edge (Siemens Healthineers) and a Somatom Force (Siemens Healthineers). The tube current modulation system (CareDose 4D) was used for all CT acquisitions and the automated selection of the kVp system (Care kV) for most acquisitions. All raw data were reconstructed using the ADMIRE algorithm.

### Dedicated software

For each CT acquisition of each pregnant patient included, the FDs were calculated independently of the doses to the uterus and/or FDs calculated by the medical physics team during conventional management.

In the present study, FDs were computed with four organ dose calculation software packages. First of all, VirtualDose-CT (Virtual Phantom Inc), which falls into the SaS (Software as Service) category and can be used via a web platform [13]. This software has been used in our institution since March 2023 and uses a library of 25 computational phantom models of real anatomy (Boundary Representation Phantoms), including three pregnant phantoms (3 months, 6 months, and 9 months; Table 1) [14]. The choice of pregnant phantom among these three phantoms is user-defined and involves a calculation method based on Monte Carlo simulation to calculate organ doses on voxelated computational phantoms [13].

**Table 1:** Characteristics of the phantoms used for the four dedicated software packages

Software	Phantom	Weight	Height	BMI	Number of CT acquisitions
	Type	(kg)	(m)	(kg/m^2^)	
VirtualDose-CT or fetaldose.org	3 months	61.9	1.63	23.2	34
	6 months	66.6	1.64	24.9	28
	9 months	72.4	1.64	27.1	7
Duke Organ Dose	pt98_3wks	108.4	1.72	36.6	8
	pt98_6wks	108.6	1.72	36.7	3
	pt98_8wks	108.7	1.72	36.7	6
	pt98_10wks	108.8	1.72	36.8	5
	pt98_15wks	110.0	1.72	37.2	7
	pt98_20wks	111.2	1.72	37.6	7
	pt98_25wks	112.8	1.72	38.1	14
	pt98_30wks	113.6	1.72	38.4	7
	pt98_35wks	116.3	1.72	39.3	2
	pt98_38wks	117.4	1.72	39.7	1
	vf50_6wks	64.1	1.63	24.1	1
	vf50_10wks	64.4	1.63	24.2	2
	vf50_15wks	66.1	1.63	24.9	2
	vf50_20wks	67.6	1.63	25.4	1
	vf50_30wks	72.3	1.63	27.2	3
CODE	0–7 weeks	56	–	–	8
	8–12 weeks	56	–	–	16
	13–25 weeks	61	–	–	21
	26–40 weeks	63	–	–	24

The second software package, Duke Organ Dose, was developed by Duke University and is available as an optional module in the DACS (dose archiving and communication system) DoseWatch (GE Healthcare). This software package uses computational phantoms from a library of 160 XCAT anthropomorphic phantoms, including 5 models of pregnant women (pt_76 (BMI: 28.6 kg/m^2^), pt_86 (BMI: 18.2 kg/m^2^), pt_98 (BMI: 35.5 kg/m^2^), pt_162 (BMI: 30.7 kg/m^2^), and vf_50 (BMI: 24.1 kg/m^2^)) with ten gestational ages (3, 6, 8, 10, 15, 20, 25, 30, 35, and 38 weeks) [16–18]. For pregnant patients, the choice of phantom depends on the number of weeks of pregnancy (WP) entered by the user, the patient’s morphology based on the CT acquisition images, and the positioning of the different anatomical regions on the radiographic positioning image. The anatomical regions (thorax, abdomen, pelvis, etc.) are automatically detected and can be adjusted manually by the user. As with VirtualDose-CT, organ doses are calculated using Monte Carlo simulations based on the methodology defined by Tian et al [26].

The third software package was fetaldose.org, a free web-based tool (www.fetaldose.org) developed by the University of Zurich [21]. This software uses the same three hybrid pregnant phantoms (3 months, 6 months, and 9 months; Table 1) used with VirtualDose-CT software and developed by Xu et al [10]. As for VirtualDose-CT, the choice of pregnant phantom among these three phantoms is user-defined, and the software uses a calculation method based on Monte Carlo simulations to calculate organ doses on voxelated computational phantoms [21]. However, to take into account the patient’s morphology, the FD calculated in the hybrid pregnant phantom is corrected by considering the patient’s maternal circumference.

The last dedicated software package was COnceptus Dose Estimation (CODE), which is a free web-based software tool (http://embryodose.med.uoc.gr/) developed by the Department of Medical Physics of the University of Crete [19, 20]. This software uses four mathematical phantoms representing the average pregnant individual during the 1st post-conception weeks (0–7 weeks), the 1st trimester (8–12 weeks), the 2nd trimester (13–25 weeks), and the 3rd trimester (26–40 weeks) of gestation. Phantoms were generated using Body Builder software, and the abdominal circumference of these phantoms was 88.7 cm, 88.7 cm, 102.3 cm, and 108.2 cm, respectively. The cumulative normalized FD was calculated in the phantom using the Monte Carlo-N-particle transport code. It was then corrected by taking into account various parameters such as correction factors for fetus depth (0–12 WP) and abdominal maternal circumference and for the specific CT scanner used for the examination (CTDI_free-in-air_, kVp, beam collimation, CTDI_vol_ of the CT acquisition) to obtain FD of the specific CT acquisition [19, 20].

### Data recorded

To calculate FDs with the four different software packages, different data and parameters were retrieved or measured.

For each patient included, the age, body mass index (BMI), and number of WP were retrieved from the picture archiving and communication system (PACS; Agfa Healthcare).

For each CT acquisition, the tube voltage (kVp), mean effective tube current-time product (mAs), and volume CT dose index (CTDI_vol_) were collected from the dose reports in the PACS. The table-feed per rotation, pitch, reconstructed slice thickness, beam collimation, and *z*-axis positions from the start and end of the acquisition were retrieved directly from the DICOM information of the images for each CT acquisition.

For patients between 1 WP and 12 WP, the depth of the fetus was measured on the CT image located in the center of the uterus, from the patient’s anterior abdominal area. For all pregnant patients, the abdominal maternal circumference was also measured in the CT section containing the central area of the uterus.

### FD calculation

For the VirtualDose-CT software, the 3-month pregnant phantom was chosen for women between 1 WP and 17 WP, the 6-month phantom for women between 18 WP and 30 WP, and the 9-month phantom for women between 31 WP and 39 WP. For each CT acquisition, the anatomical exposure area was manually placed on the phantom according to the anatomical landmarks in the CT images and the length of the exposed area using the *z*-position of the first and last slices. Then, after selecting the CT system (manufacturer and model) and the bowtie filter, the kVp, beam collimation, and pitch were entered. Finally, the tube current modulation was selected and the mAs were adapted to obtain the CTDI_vol_ of the CT acquisition. For each CT scan acquisition, the total FDs calculated were recorded.

For Duke Organ Dose and for each CT acquisition, as the dosimetric information (kVp, pitch, beam collimation, CTDI_vol_, mAs modulation along the *z*-axis, etc.) and the start and end positions of the exposed area are directly available in the CT images on the DACS, only the number of WPs was entered and the proposed anatomical zones adjusted. The pregnant phantom was automatically selected for each CT acquisition (Table 1) and the FD (fetus-body) was recorded.

For fetaldose.org, as the phantoms are the same, the choice of a pregnant phantom according to the number of WP was made in the same way as for VirtualDose CT. For each CT acquisition, the kVp, the CTDI_vol_, and the abdominal maternal circumference were entered. Then, the length of the exposed area was defined using the same methodology as that defined for VirtualDose-CT. The FD (Radiation dose to the fetus) calculated was recorded.

For CODE, the “0–7-week” pregnant phantom was chosen for women between 1 WP and 7 WP, the “8–12-week” phantom for women between 8 WP and 12 WP, the “13–25-week” phantom for women between 13 WP and 25 WP and the “26–40-week” phantom for women between 26 WP and 40 WP. For each CT acquisition, the mAs, kVp, pitch, and maternal abdominal circumference were entered. For women between 1 WP and 12 WP, the fetal depth was also entered. Next, the CTDI_free-in-air_ (mGy/100 mAs) available in the CT datasheet according to the kVp and the beam collimation, and the CTDI_w_ (mGy/100 mAs) of the acquisition were entered. Finally, the length of the exposed area was defined using the same methodology as that defined for VirtualDose-CT, and the FD (embryo dose) calculated was also recorded.

### Statistical analysis

Statistical analyses were performed using R software version 4.4.0. Qualitative variables are presented as numbers and percentages. Quantitative variables are presented as means and standard deviations, medians, and interquartile ranges.

Linear regression analyses were carried out to assess the association between the BMI or the number of WP of the patients and those of the phantoms selected by Duke Organ Dose software. They were also used to evaluate the association between the CTDI_vol_ values and FD values computed by each software package.

Differences in FDs measured by the different software packages were analyzed via a mixed linear regression model, explaining FD according to the software package, adjusted for age, BMI, WP, circumference, and CTDI_vol_. A random intercept for each patient was also included to take into account repeated measurements with different software. An additional analysis was also made via the intra-class correlation coefficient to evaluate the agreement between software packages.

Finally, the parameters associated with the FD calculated were analyzed using a linear regression model for each measurement mode, and then taking all the measurement modes into account in a mixed linear regression model. The estimates and their 95% confidence intervals are presented. Due to multiple comparisons and the greater risk of falsely significant results at the initial 0.05 thresholds, a difference between groups was considered to be statistically significant at a more restrictive *p*-value of less than 0.01.

## Results

### Patients

**Fig. 1:**
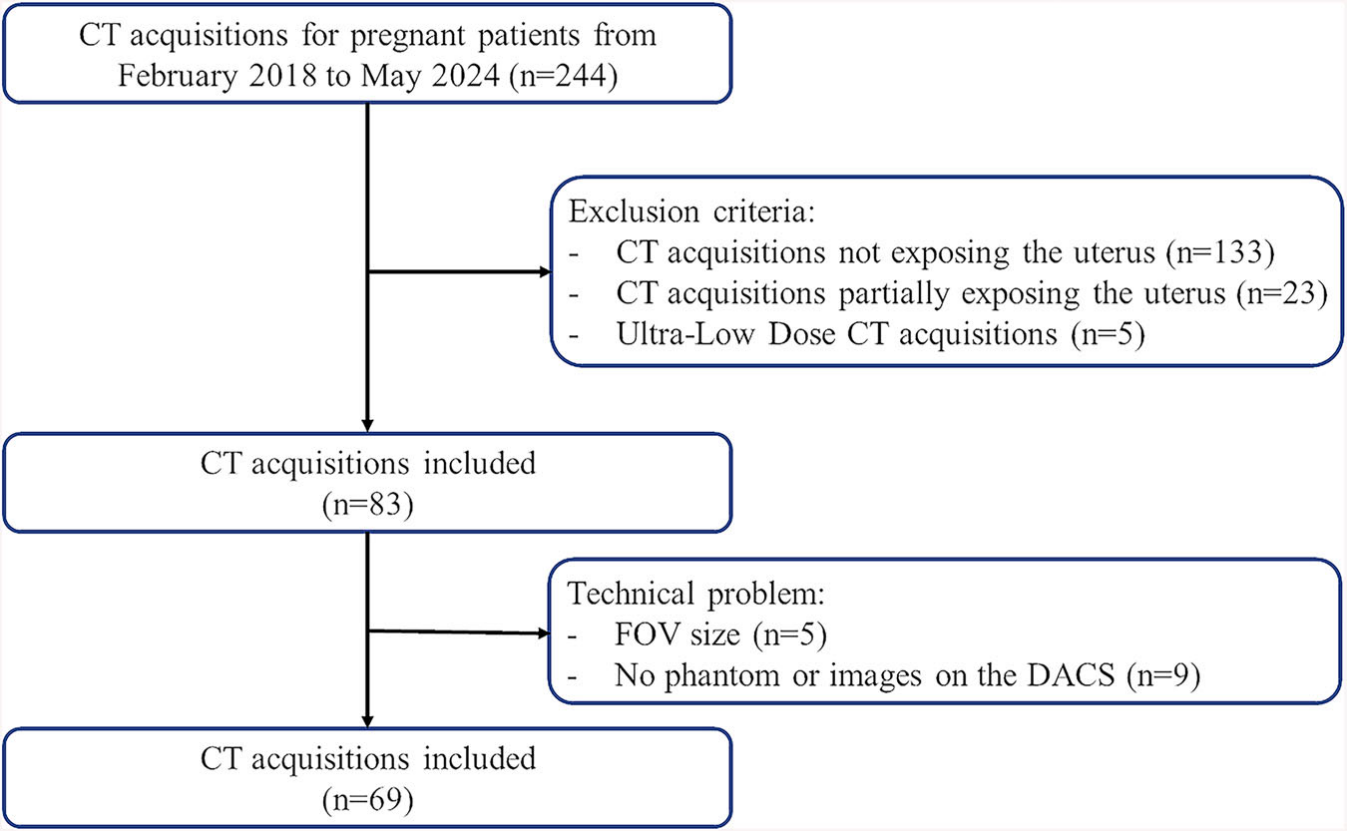
Study flow chart

A total of 51 pregnant patients were included during the study period (Fig. 1). The patients’ mean age was 30.3 ± 6.0 [19–42]-years-old, and their mean gestational stage was 19.3 ± 9.8 [1–38] WP. The mean BMI was 27.5 ± 6.3 [17–52] kg/m^2^.

A total of 69 CT acquisitions were assessed, 62 abdomen–pelvis and 7 for thorax–abdomen–pelvis CT acquisitions (Table 2). The mean number of acquisitions per pregnant patient was 1.4 ± 0.7 [1−4]. For all CT acquisitions, the mean CTDI_vol_ was 6.77 ± 3.04 [2.34–15.64] mGy on the CT report (Table 2).

**Table 2:** Patient data

	Abdomen–pelvis	Chest–abdomen–pelvis	Total
Number of patients	48	7	51
Number of acquisitions	62	7	69
Age (year)	30.1 ± 5.8 [19–41]	31.5 ± 7.0 [24–42]	30.3 ± 6.0 [19–42]
WP	19.1 ± 9.9 [1–38]	15.1 ± 11.7 [1–32]	19.3 ± 9.8 [1–38]
BMI (kg/m^2^)	27.3 ± 6.2 [17–52]	26.2 ± 7.4 [18–36]	27.5 ± 6.3 [17–52]
Number of CT acquisitions	1.3 ± 0.7 [1–4]	1.0 ± 0.0 [1; 1]	1.4 ± 0.7 [1–4]
CTDI_vol_ (mGy)	6.88 ± 3.06 [2.3–15.6]	5.86 ± 2.89 [3.4–11.0]	6.77 ± 3.04 [2.3–15.6]

Values are expressed as means ± standard deviations [range]

### Phantoms used

For VirtualDose-CT and fetaldose.org, the 3-month pregnant phantom was selected for 34 CT acquisitions, the 6-month phantom for 28 CT acquisitions, and the 9-month phantom for 7 acquisitions (Table 1). For each phantom, the mean number of WP and the patients’ BMIs were 8.6 ± 4.8 [1–17] WP and 25.8 ± 5.7 [17.3–35.1] kg/m^2^, 24.3 ± 3.4 [18–30] WP and 26.9 ± 5.7 [19.4–39.4] kg/m^2^ and 33.1 ± 2.5 [31–38] WP and 30.6 ± 9.8 [22.1–51.6] kg/m^2^, respectively.

**Fig. 2:**
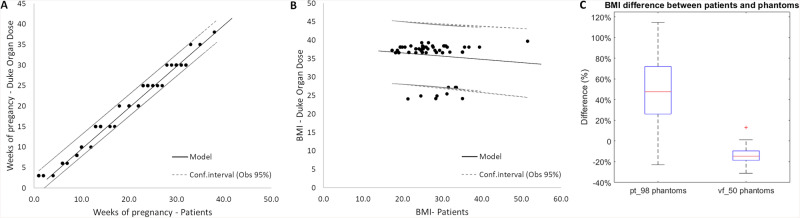
**A** Number of WP of the phantoms used with Duke Organ Dose software according to the patient’s real stage of pregnancy (linear regression). **B** BMI of the phantoms used with Duke Organ Dose software according to the patient’s real BMI (linear regression). **C** Differences in BMI between the BMIs of the two phantoms used with Duke Organ Dose software and the patient’s real BMI

For Duke Organ Dose, two of the 5 available phantoms were selected by the software; pt_98 (BMI ranging from 36.6 kg/m^2^ to 39.7 kg/m^2^) for 60 CT acquisitions and vf_50 (BMI ranging from 24.1 kg/m^2^ to 27.2 kg/m^2^) for 9 CT acquisitions (Table 1). The correlations between the patients’ number of WP and those of the selected phantoms were excellent for phantoms pt_98 and vf_50 with a linear regression coefficient (*R*^2^) of 0.984 and 0.989, respectively (Fig. 2A). For BMI, the correlation was poor for the vf_50 phantoms (*R*^2^: 0.280), for the pt_98 phantoms (*R*^2^: 0.058) and for both phantoms (*R*^2^: 0.017) (Fig. 2B). Overall, the BMIs of the selected pt_98 phantoms were higher than those of the pregnant patients (median difference 47.6% (26.4%; 71.2%)) and the opposite for the vf_50 phantoms (median difference −14.7% (−18.6%; −13.1%)) (Fig. 2C).

For CODE, the “0–7 weeks” pregnant phantom was selected for 12 CT acquisitions, the “8–12 weeks” phantom for 13 CT acquisitions, the “13–25 weeks” phantom for 29 CT acquisitions and the “26–40 weeks” phantom for 15 CT acquisitions (Table 1).

### Fetal doses

Table 3 shows the FD values obtained with the four software programs for all CT acquisitions, but also according to the WP and the type of CT acquisitions. For all CT acquisitions, the highest FD values were found with VirtualDose-CT and the lowest with fetaldose.org.

**Table 3:** FD per CT acquisition obtained with VirtualDose CT, Duke Organ Dose, fetaldose.org, and CODE for all CT acquisitions and for the defined subgroups of CT acquisitions and WP

		FD (mGy)			
		VirtualDose-CT	Duke Organ Dose	fetaldose.org	CODE
Subgroups type of CT acquisitions	Abdomen–pelvis	8.5 (6.4; 10.3)	7.7 (6.2; 9.6)	6.5 (4.9; 7.8)	7.2 (5.1; 8.8)
	Chest–abdomen–pelvis	10.2 (7.9; 11.8)	8.4 (4.7; 12.4)	6.2 (4.7; 6.7)	4.9 (3.8; 9.0)
Subgroups WP	From 1 to 17 weeks	8.4 (6.3; 10.6)	7.0 (5.8; 10.5)	6.8 (4.9; 8.1)	7.2 (4.9; 8.7)
	From 18 to 30 weeks	8.6 (6.5; 10.6)	8.2 (6.5; 9.2)	5.9 (4.6; 6.8)	5.7 (4.4; 8.0)
	From 31 to 40 weeks	10.2 (8.8; 10.2)	11.9 (8.7; 12.6)	6.6 (6.0; 7.0)	8.8 (8.1; 9.6)
All		8.6 (6.8; 10.3)	7.7 (6.1; 9.7)	6.3 (4.9; 7.6)	7.1 (4.6; 8.8)

Values are expressed as medians (1st quartile; 3rd quartile)

**Fig. 3:**
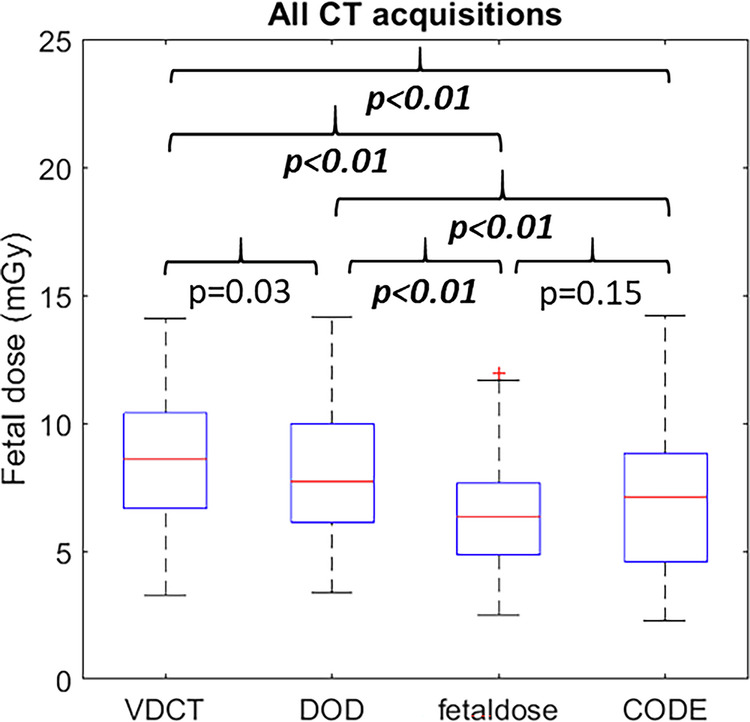
Fetal doses calculated with VirtualDose-CT, Duke Organ Dose, fetaldose.org, and CODE for all CT acquisitions. A *p*-value of less than 0.01 was considered significant

The differences between each software calculation were significant (*p* < 0.01), except between VirtualDose-CT and Duke Organ Dose (−6.3% (−16.3; 14.6%); *p* = 0.025) and between CODE and fetaldose.org (6.9% (−15.8; 27.3%); *p* = 0.15) (Fig. 3).

The median relative differences in percentage between FD calculated by the four software packages are depicted in the Supplementary Material file (Table 1-SM). The differences in FDs calculated between each software according to the WP (Fig. 1-SM) and the type of CT acquisitions (Fig. 2-SM) are depicted in the Supplementary Material file.

The agreement on calculated FD values between the software packages is depicted in the Supplementary File. The agreement between the software packages was poor (ICC < 0.50), except between VirtualDose-CT and CODE and between fetaldose.org and CODE (Table 1-SM).

**Fig. 4:**
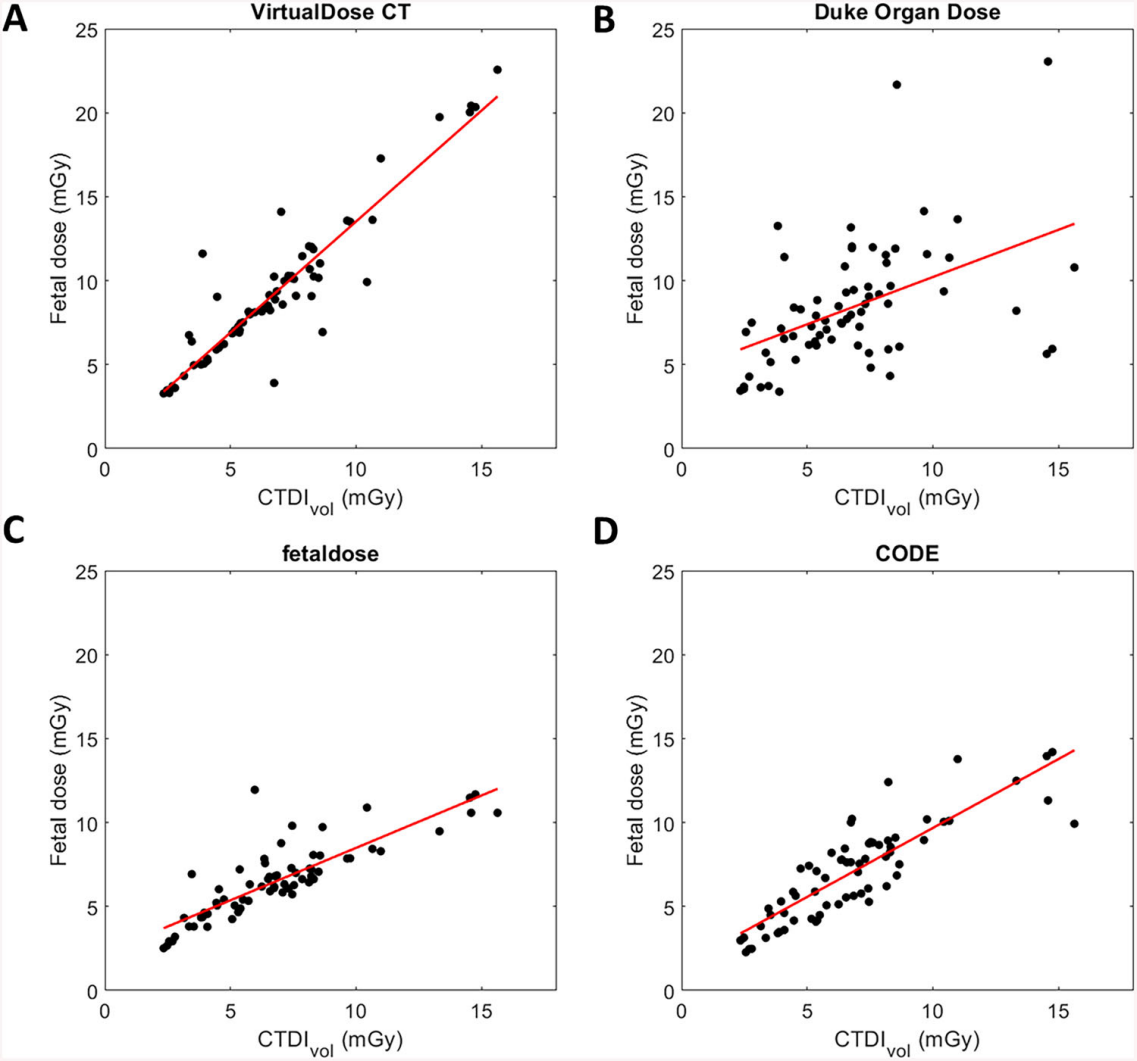
Fetal doses calculated with VirtualDose-CT (VDCT; **A**), Duke Organ Dose (DOD; **B**), fetaldose.org (**C**), and CODE (**D**) according to the CTDI_vol_ used (linear regression)

The correlation between CTDI_vol_ and FD values was good for VirtualDose-CT (*R*^2^: 0.863), fetaldose.org (*R*^2^: 0.716), and CODE (*R*^2^: 0.731) but poor for Duke Organ Dose (*R*^2^: 0.220) (Fig. 4).

### Multivariate analysis

The multivariate analysis of FD calculated by each type of software and adjusted for different characteristics is depicted in Table 4. No significant differences were found in FDs according to BMI, age, circumference, and WP (except for fetaldose.org; *p* < 0.001). Statistical differences were found for FDs according to CTDI_vol_ for all software, except for Duke Organ Dose (*p* = 0.079).

**Table 4:** Multivariate analysis of the association between the various parameters and dose to fetus as calculated by each software package

	VirtualDose CT		Duke Organ Dose		fetaldose.org		CODE	
Characteristics	beta [CI 95%]	*p*-value	beta [CI 95%]	*p*-value	beta [CI 95%]	*p*-value	beta [CI 95%]	*p*-value
BMI	0.05 [−0.07, 0.18]	0.4	0.14 [−0.11, 0.39]	0.3	−0.01 [−0.08, 0.05]	0.7	−0.02 [−0.15, 0.09]	0.7
Age	−0.02 [−0.90, 0.05]	0.6	0.03 [−0.11, 0.17]	0.7	−0.01 [−0.05, 0.02]	0.5	0.01 [−0.06, 0.07]	0.8
Week of pregnancy	−0.01 [−0.05, 0.03]	0.6	0.08 [0.00, 0.16]	0.057	−0.07 [−0.09, −0.05]	< 0.001	−0.01 [−0.05, 0.02]	0.5
Circumference	0.04 [−0.04, 0.09]	0.5	−0.03 [−0.16, 0.10]	0.7	−0.05 [−0.08, −0.02]	0.004	−0.03 [−0.09, 0.03]	0.3
CTDI_vol_	1.20 [0.95, 1.40]	< 0.001	0.39 [−0.04, 0.82]	0.079	0.84 [0.73, 0.95]	< 0.001	0.97 [0.76, 1.2]	< 0.001

### Particular case of one pregnant patient

During the inclusion period, one patient (30-years-old, BMI 35.1 kg/m^2^ and 12 WP) underwent three CT examinations, including 4 CT acquisitions of the abdomen–pelvis and one CT acquisition of the chest–abdomen–pelvis. Table 5 shows the cumulative FD obtained with the various dedicated software packages. The highest FD was obtained with VirtualDose-CT and the lowest with Duke Organ Dose.

**Table 5:** Total FD for a pregnant patient (30-years-old, BMI 35.1 kg/m^2^ and 12 WP)  exposed to 5 CT acquisitions as obtained by the four different software packages

Software	Phantom(s) used	BMI (kg/m^2^)	FD (mGy)
VirtualDose CT	3-months	23.2	77.92
Duke Organ Dose	pt_98_10 weeks	36.8	34.00
fetaldose.org	0–3 months	23.2	58.34
CODE	8–12 weeks	–	54.46

## Discussion

In this study, the radiation doses delivered to the fetus according to four different dedicated software packages were calculated and compared for 69 CT acquisitions fully exposing the fetus. For all software, it was possible to use phantoms that took the stage of pregnancy into account.

The results of this study confirm that the differences between the four software packages led to differences in the FDs they calculated. For similar CTDI_vol_ values per CT acquisition, the highest FDs were obtained with VirtualDose-CT and the lowest with fetaldose.org. These differences in fetal doses calculated between the four software packages were significant, except between VirtualDose-CT and Duke Organ Dose and between fetaldose.org and CODE. These variations can be explained by the difference in phantoms and calculation methods used by each software. For an accurate FD calculation, it is important to use a phantom that simulates the pregnancy stage as closely as possible to that of the patient and takes the patient’s anatomy and morphology into account. In VirtualDose-CT, three pregnant phantoms are available [13, 14]. Despite the increase in BMI between the three pregnant phantoms, they remained far from the reality of the BMIs of patients studied, as most of the time the phantoms’ BMIs were lower. To take account of the patient’s morphology, the fetaldose.org software, which uses the same phantoms as VirtualDose-CT, corrected the calculated FD by taking into account the patient’s maternal circumference [10, 21]. Duke Organ Dose has a larger phantom library of pregnant patients (five pregnant phantoms with ten gestational stages), which means it can match a patient’s morphology more closely [16–18]. However, in this study, only two of the five available pregnant phantoms were selected by the software. The correlation between the BMIs of the two phantoms and those of the patients was poor, and most of the time, the BMIs of the phantoms were too high. Compared with the other three software packages, CODE does not use hybrid or computational phantoms, but mathematical phantoms. The morphology of the phantom selected according to the number of WP is adapted according to the fetus’ depth (for WP ranging from 0 to 12) and maternal abdominal circumference [19, 20]. These differences in morphology between the phantom used in each software and the pregnant patient (BMI) have an impact on FD calculation, particularly on differences in attenuation of the photon beam and distribution of the radiation dose. It should also be noted that there are other differences between these two software packages. With VirtualDose-CT, fetaldose.org, and CODE, the input data is limited to data entered by the user and does not take into account the real modulation of mAs during CT acquisition or the exact position of the exposure area. In addition, as the acquisition images are available on the DACS, Duke Organ Dose can access and use this information directly to calculate FD. Finally, the calculation methods and the way in which the FD is calculated also differ between software packages. With VirtualDose-*CT, FD is calculated using “Fetus-Total”, whereas Duke Organ Dose uses “Fetus-Body” and CODE uses “Embryo Dose”. So far, no studies have been found in the literature to compare these items.

In some cases, the choice of calculation software used may affect the care of the pregnant patient and the fetus. In the particular case presented, which initiated this study, as none of the four software packages used in the study were accessible or known by the medical physics team (all the CT acquisitions were performed in March 2023), the dose to the uterus was calculated using CT-Expo software with a single standard phantom [27]. The cumulative dose to the uterus calculated with CT-Expo (e.g., 104.2 mSv) exceeded the dose limit at 100 mGy in utero defined by the ICRP. Based on this result alone, exceeding the threshold may justify termination of pregnancy based on the radiological risk alone. However, the FDs calculated for these other four software packages with appropriate phantoms to take the stage of pregnancy into account—in this case, 3 months—led to values below this threshold. The differences in values obtained by the four software packages were directly related to the differences in morphology and composition of the phantoms used, as explained above. This was especially true in this particular case, where the patient had a BMI similar to that of the phantom used with Duke Organ Dose (lowest cumulative FD) but far from that of the phantom used with the other three software packages, especially VirtualDose CT (highest cumulative FD). The values obtained with fetaldose.org and CODE, which are between those of the other two software packages, can be explained by the fact that the patient’s circumference and the depth of the fetus (only for CODE) are taken into account to calculate the FD. Based on the analysis of this case and, as there is no official standardized method, the results of the present study show that it is essential to use software with phantoms as faithful as possible to the patient’s morphology and stage of pregnancy. As the accuracy of the software cannot be assessed, the closer the phantom used is to the reality of the patient and the closer the parameters used are to those used during CT acquisition, the better the FD calculations.

This study has certain limitations. Firstly, no reference method (measurements on patients, Monte Carlo simulation) was available to assess the accuracy of results obtained in terms of FD with the four software packages. CT imaging exposure of an anthropomorphic phantom with dosimeters in the area of uterine area or Monte Carlo simulation-derived dose estimates could act as ground truth. However, our institution does not currently have the equipment and/or tools to make these assessments. The use of a reference method would have strengthened the results of the study and helped to determine which software best corresponds to the reality for calculating the FD. Secondly, to facilitate comparisons between the different software packages, particularly in relation to Duke Organ Dose, FDs were calculated based on the average mAs—not the maximum mAs—in the uterus, as may be the case for certain studies. Thirdly, only the FDs of acquisitions directly exposing the fetus were compared in the present study. A complementary study could be performed to compare the results obtained for acquisitions partially exposing the uterus (lumbar spine, dorsal spine, chest–abdomen, abdomen, pelvis, etc.). However, for this type of CT acquisition, the risk of approaching the 100 mGy limit could be even lower than for acquisitions exposing the whole fetus. Finally, this study reflects the practices of only one center with 12 years’ experience of thorough optimization processes and the presence of medical physicists.

In conclusion, the choice of software used has an impact on the calculation of radiation doses delivered to the fetus. The results of the present study show that the 100 mGy limit cannot be reached by a single CT examination, and may only be reached for extreme cases in which the pregnant patient is subjected to multiple CT examinations with the whole fetus exposed primarily.

## Supplementary information

ELECTRONIC SUPPLEMENTARY MATERIALdocx

## Abbreviations

BMI: Body mass index
CT: Computed tomography
CTDI_vol_: Volume CT dose index
FD: Fetal dose
WP: Weeks of pregnancy
