# Supplementary material for: Fetal dose in pregnant CT patients: a comparison of four software packages
Source: Eur Radiol. 2025 Apr 25;35(10):6258–67. doi: 10.1007/s00330-025-11594-1 (PMC12417268; doi:10.1007/s00330-025-11594-1)

Fetal dose in pregnant CT patients: a comparison of four software packages

ELECTRONIC SUPPLEMENTARY MATERIAL

Table 1-SM. Agreement and median relative differences between fetal dose values calculated by the 4 software packages.

Agreement between:			ICC [95% CI]	Relative differences (%)
VirtualDose CT	*vs.*	Duke Organ Dose	0.41 [0.19; 0.58]	-6.3 (-16.3; 14.6)
VirtualDose CT	*vs.*	fetaldose.org	0.48 [0.02; 0.72]	-28.1 (-38.1; -13.2)
VirtualDose CT	*vs.*	CODE	0.61 [0.18; 0.80]	-23.8 (-37.1; -7.4)
fetaldose.org	*vs.*	Duke Organ Dose	0.25 [0.02; 0.45]	-27.2 (-39.3; 1.9)
CODE	*vs.*	Duke Organ Dose	0.33 [0.11; 0.52]	-12.5 (-37.0; 4.0)
fetaldose.org	*vs.*	CODE	0.75 [0.62; 0.84]	6.9 (-15.8; 27.3)

Values of relative differences are expressed as median (1st quartile; 3rd quartile).

Figure 1-SM. Fetal doses calculated with VirtualDose CT, Duke Organ Dose, fetaldose.org and CODE for all CT acquisitions for the three sub-groups of weeks of pregnancy. A p-value of less than 0.01 was considered significant.

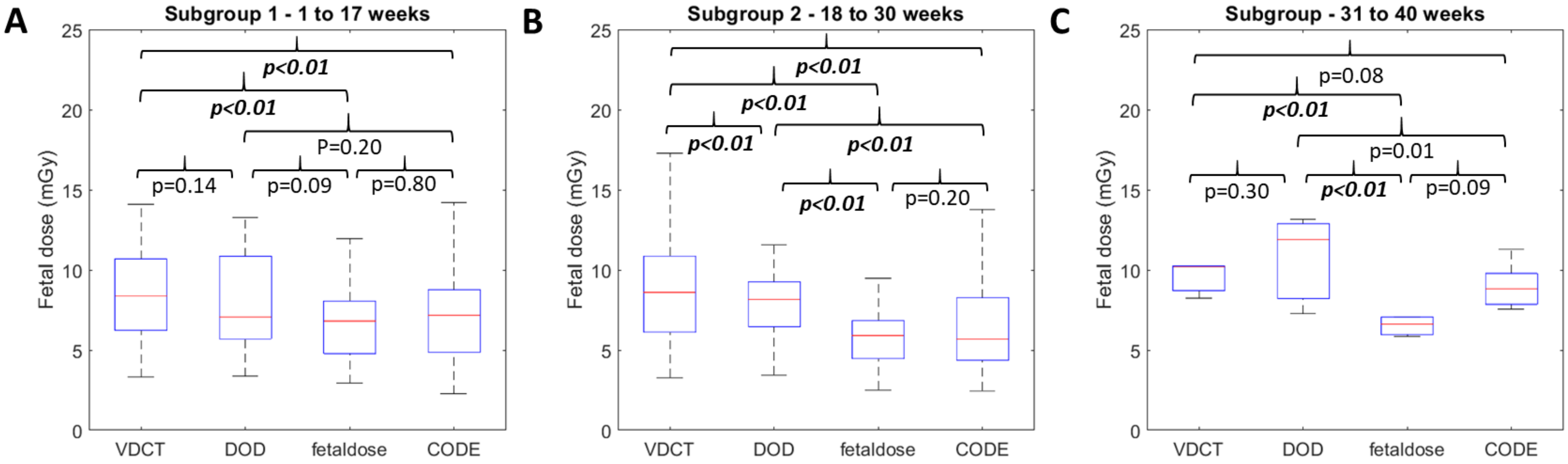

Figure 2-SM. Fetal doses calculated with VirtualDose CT, Duke Organ Dose, fetaldose.org and CODE for all CT acquisitions for the two sub-groups of CT acquisition types. A p-value of less than 0.01 was considered significant.

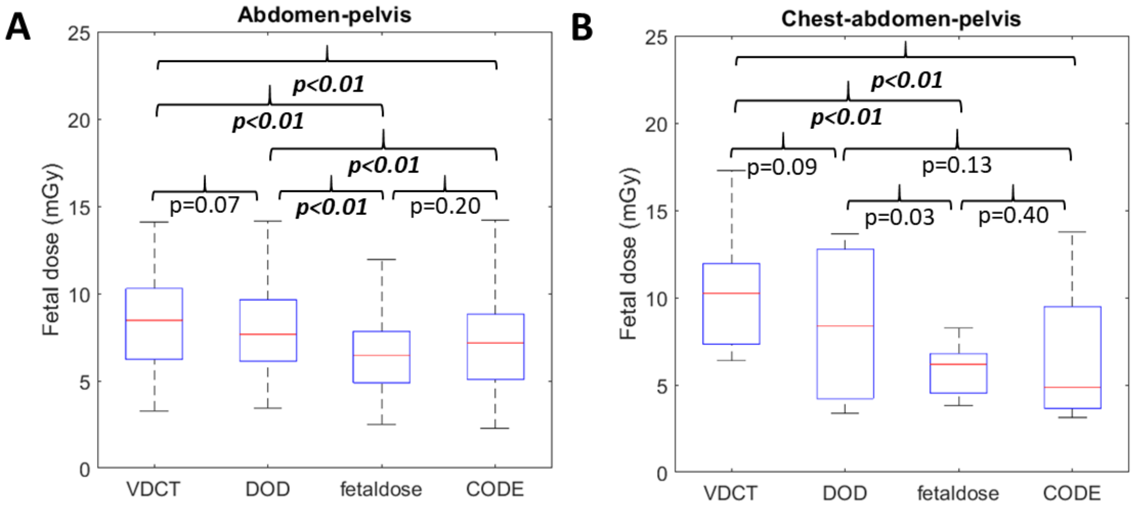

Supplement: Supplementary file 1 — ELECTRONIC SUPPLEMENTARY MATERIALdocx [file 330_2025_11594_MOESM1_ESM.pdf]
